# Compartmental analysis of metals in waterpipe smoking technique

**DOI:** 10.1186/s12889-015-1373-6

**Published:** 2015-02-18

**Authors:** Akeel T Al-Kazwini, Adi J Said, Stephanie Sdepanian

**Affiliations:** Department of Biomedical Engineering, School of Applied Medical Sciences, German Jordanian University, P.O. Box 35247, Amman, 11180 Jordan; Scientific Research Centre, Applied Science Sector, Royal Scientific Society, Amman, Jordan; Department of Pharmaceutical Engineering, School of Applied Medical Sciences, German Jordanian University, Amman, Jordan

**Keywords:** Waterpipe, Moassel, Metals, Tobacco, Filtering, Smoking machine

## Abstract

**Background:**

The number of waterpipe tobacco smokers has been increasing worldwide. Smokers can be exposed to a number of toxicants, some of which are metals. The aim of this study is to quantitatively determine if the water filtration stage of the waterpipe smoking process successfully decreases exposure to Bi, Cr, Cu, Fe, Mg, Mn, Mo, Ni, Pb, V, and U.

**Methods:**

Four samples of commercially available tobacco (Moassel) were compared in terms of the total amount of metal contained within the fresh tobacco sample and the amount of metal distributed into each compartment of the waterpipe after a smoking session.

**Results:**

For all metals analysed, the concentration of metal ‘filtered’ out during the water bubbling stage is around 3% (±1%) of the total metal.

**Conclusions:**

It can be concluded that this small fraction would not protect the user against exposure to the majority of the potentially toxic metals.

## Background

The use of a waterpipe for the purposes of smoking tobacco is an ancient tradition in many parts of the world [1]. The waterpipe bears a number of different names depending on the set-up and region of origin; some typical names are Hubbly-Bubbly, Narghile, Goza, Shisha, and Hookah. The tobacco mixtures used in waterpipes vary widely and differ from other available types of tobacco. Commonly referred to as ‘Moassel’, the mixture usually contains around 30% tobacco, and the remaining 70% is a concoction of flavourings, glycerol and sweeteners (e.g. molasses and honey) [2].

There are clear differences between waterpipe and cigarette smoking. According to the WHO report on waterpipe use, a typical session of smoking, which can last up to an hour, exposes the user to 100–200 times the volume of smoke inhaled in a single cigarette. The smoke contains a mixture of toxicants including carbon monoxide and metals. A number of the toxicants present are known carcinogens [1]. In regions where waterpipe tobacco smoking has been adopted into the culture, there is a common yet unfounded belief that waterpipe smoke is made less harmful when bubbled through water before inhalation [3,4].

Exposure to elevated concentrations of heavy metals is known to cause adverse effects to humans [5]. The use of tobacco mixtures in waterpipes may be a route of exposure to toxic metals in humans [6]. Trace elements can be taken up and accumulated by plants [7]. Therefore, the environment in which tobacco plants are grown (in terms of soil and water) significantly affects the concentrations of trace metal elements in the leaves. There have been a number of studies investigating the exposure of waterpipe users to a variety of harmful substances. Some examples include polyaromatic hydrocarbons, volatile organic compounds, carbon monoxide, nicotine, particulate matter, volatile aldehydes, tobacco specific nitrosamines, and radionuclides [8-17]. However, few studies have focused on the study of metals in waterpipe tobacco [2].

The objective of this study is to determine whether the ‘water filtration’ stage of the waterpipe smoking process removes a significantly large fraction of the total metal contained in the fresh samples of tobacco. Likewise, to investigate if the water filtration process differs among the metals studied (namely Bismuth (Bi), Chromium (Cr), Copper (Cu), Iron (Fe), Magnesium (Mg), Manganese (Mn), Molybdenum (Mo), Nickel (Ni), Lead (Pb), Vanadium (V), and Uranium (U)) or between four commercially available waterpipe tobacco samples with different flavours and colours. Establishing if a significant proportion of the metal available is trapped in the water vessel on completion of the smoking session will also help determine if compartmental analysis of new tobacco samples is necessary. Alternatively, if simply a measure of the total metal and residue concentration is required to reasonably determine any potential risk from toxic metals.

## Methods

Four samples of tobacco were compared in terms of the total amount of metal in the fresh tobacco sample and the amount of metal distributed into each compartment after a smoking session. The compartments examined were; the main smoke stream, the water in the vessel (through which the smoke was bubbled), the filter, and finally the ash residue. The ash residue is what remains of the burnt tobacco at the cessation of the smoking session. The mixtures of tobacco used are of the ‘Moassel’ variety and for the purposes of clarity will be referred to as ‘waterpipe tobacco’ throughout this paper. Despite regional variation in the method of use and structure of waterpipes available the one used during this study is commonly referred to as ‘Shisha’ and is described in the methodology section, the term ‘waterpipe’ used will specifically refer to the procedure described therein.

### Waterpipe tobacco samples

Four samples of waterpipe tobacco, which are commercially available on the Jordanian market, were selected for use in this study. The samples were purchased from the Jordanian local market in July 2012 and represent the most popular brands and flavours. The brands used were either made locally in Jordan or imported from the United Arab Emirates [16]. The samples were labelled using code numbers detailing the flavour and colour (Table 1).

**Table 1:** **Labelling of the tobacco samples**

**Sample code**	**Flavour**	**Colour**
01APPRD	Apple	Red
06GRPBR	Grape	Brown
13LWMBR	Lemon and mint	Brown
12LWMBR	Lemon and mint	Brown

List of four analysed tobacco samples each with flavour and colour specified.

### Experimental procedure

The selected samples were analysed in a two-step process. The first step involved determining the total metal concentration using an acid digestion. For the second step, a shisha (waterpipe) smoking machine was used to analyse the concentration of metals in the four compartments. The amount of metal in the smoke stream was calculated as the difference between the total metal in the waterpipe tobacco and the sum of metal in the water vessel and the ash residue. The values reported as the concentration of metal in smoke are used as a measure of the metal that is not filtered by the water and has a smaller particle size than that of the filter but is still available for contact with the user, as it is not trapped by the remaining residue. The 01APPRD tobacco sample was used as an independent quality control sample due to the unavailability of suitable reference material with a comparable matrix. The metals analysed were Bi, Cr, Cu, Fe, Mg, Mn, Mo, Ni, Pb, V, and U. All chemical analysis was performed in the Royal Scientific Society Laboratories in Amman, Jordan.

### Shisha (waterpipe) smoking machine

**Figure 1:**
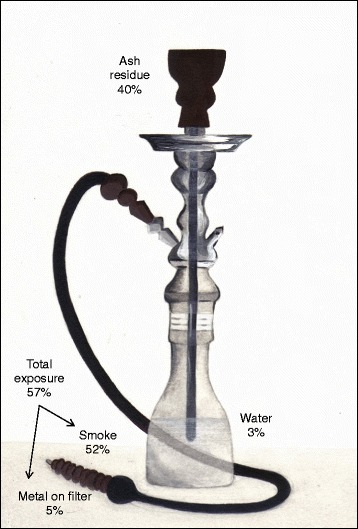
**Sketch illustrates the ‘Shisha’ set-up including the ‘head’ for containing the tobacco, the plastic hose for drawing smoke contains the filter and the water vessel through which the smoke is bubbled.** The metal concentration distribution through the compartments of a waterpipe is also included. The values are averages of all metals (Bi, Cr, Cu, Fe, Mg, Mn, Mo, Ni, Pb, V, and U) and for four commercially available waterpipe tobacco samples. *Sketch commissioned by authors* [16]*.*

**Figure 2:**
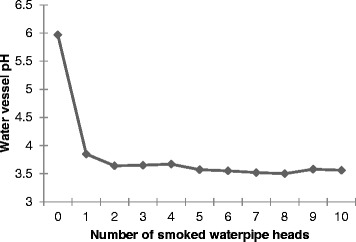
**pH profile of the water vessel as a function of the sum of smoked waterpipe tobacco heads.** Following the consumption of the second tobacco head, the pH stabilizes at around a value of 3.5.

Smoking was reproduced by connecting a Borgwaldt Shisha Smoker machine (model number 80220100) to a standard waterpipe (Borgwaldt KC) using a plastic hose (Figure 1). Waterpipe tobacco was placed into the waterpipe head and covered with perforated aluminium foil. A commonly locally used charcoal (Royal, China) was lit by means of cooking gas and placed above the foil in order to start the smoking process. The charcoal was placed on top of the foil to avoid direct contact with the tobacco such that the mixture was heated to a high temperature but not burned. The temperature under the aluminium foil and in the middle of the waterpipe tobacco head was found to be 250 and 130°C, respectively. The temperatures of the downstream smoke (after the waterpipe head and before the water vessel) and the upstream smoke (after the water vessel and before the mouth tip) were found to be 72 and 32°C, respectively. The smoke drawn through the plastic hose was then bubbled through water, thus cooling the smoke temperature by more than half. This is in accordance with a number of other studies supporting the role of the water vessel as a cooling stage for the smoke generated [4,10,18]. The water vessel temperature was increased from the ambient temperature (22°C) up to 24°C during the smoking period. Whatman filter paper with a 90 mm diameter and a pore size of 0.45 μm was placed at the end of the plastic hose to filter the emerging smoke, which allowed the fraction of smoke with particle size of 0.45 μm and above to be determined. Each smoking session completely consumed 22 g of waterpipe tobacco. The duration of each inhale was 2.6 s with a volume of 530 ml at a rate of 3 inhales (puffs) per minute (at intervals of 17 s). These parameters were selected according to the draft of the ISO/TC 126/SC standard. Ten consecutive smoking sessions were used for each sample. This equated to a total tobacco weight of 220 g analysed for each sample; the water volume in the vessel was 750 ml and was not replaced during the ten sessions. This was done in order to ensure detectable metal concentrations and a more representative sample. The pH of the water vessel before smoking was found to be 6.0 and after ten successive smoking sessions was 3.6 (Figure 2). After sampling, the water vessel samples were acidified using 1.0 ml of analytical grade concentrated nitric acid and the pH value was measured as 1.8. Five filters were replaced for each smoking session to sustain the least possible suction resistance and that was done by maintaining a pressure drop of less than 1500 Pa between the head of waterpipe and the suction device. The filter replacement was done during the 17 s interval of puff frequency.

### Acid digestion of samples

Fresh waterpipe tobacco samples, filter paper samples, and ash residue from the smoking machine were digested using a nitric acid/hydrogen peroxide solution. Ultra-pure grade 65% nitric acid and 30% pure grade hydrogen peroxide (Scharlau Chemie S.A, Spain) was used. Prior to digestion both reagents were further purified through sub-boiling distillation in a Teflon still (MLS GmbH, Germany). For each acid digestion analysis an acid washed 100 ml tall form quartz digestion beaker covered with watch glass was used and by adding various volumes (25–30 ml) of HNO_3_ and 3–5 ml of H_2_O_2_ (added in five steps). The duration of the digestion was the time required to obtain a clear solution during continuous heating at about 80°C. For the acid digestion analysis, 10.0 g of homogenised waterpipe tobacco was used. Ash residue analysis used the residue remaining after 22.0 g of fresh tobacco was used during a smoking session. Finally, the amount of metal remaining on the filters used in the smoking machine was analysed by digestion. Measurement accuracy and quality control were maintained for the digestion procedure through digestion of certified and reference standard material in the form of tealeaves (Certified reference no. 23, National Institute for Environmental Studies (NIES)) and wheat flour (Certified reference no. 1567a, National Bureau of Standards, (NIST)) for Cu, Fe, Mg, Mn, Mo, and Ni. Whereas for Cr, Pb, V, U, and Bi, an in-house standardized was formulated by spiking the matrix of Ma’assel sample no. 01APPRD with three different concentrations of aqueous standard multi-element solution, following standard addition methodology. It was concluded that the digestion was accurate with deviation levels lower than 7% of the relative standard deviation (RSD) from the assigned figures (Table 2).

**Table 2:** **Results of the measurement of accuracy for the certified, reference, and in-house standardized samples using the standard addition method**

**Element**	**Certified, reference and in-house standardizes value (mg/kg)**	**Obtained value (mg/kg)**	**% RSD** ^**1**^
**Cu**	2.1 ± 0.2^a^	2.2	4.8
**Fe**	14.1 ± 0.5^a^	14.4	2.1
**Mg**	400 ± 20^a^	409	2.3
**Mn**	9.4 ± 0.9^a^	10.0	6.4
**Mo**	0.48 ± 0.03^a^	0.45	6.3
**Ni**	7.89 ± 0.57^b^	7.73	2.0
**Cr**	48.6^c^	47.0	3.2
**Pb**	1.9^c^	2.0	4.2
**V**	1.6^c^	1.5	6.3
**U**	0.193^c^	0.203	5.2
**Bi**	0.037^c^	0.035	5.4

^1^% Relative Standard Deviation (%RSD) = (absolute (reference – obtained)/CRM) × 100.

^a^Wheat flour (NIST 1567a).

^b^Tea leaves (II NIES No. 23).

^c^In-house standardization achieved using the standard addition method with the 01APPRD tobacco sample. This sample was also used as an independent quality control sample due to the unavailability of suitable reference material with a comparable matrix.

### Sample analysis for metal concentrations

All acidified water samples from the smoking machine and acid digestion samples were analysed using Inductively Coupled Plasma Mass Spectrometry (ICP-MS) Elan DRCe 9000 (Perkin-Elmer-Sciex, USA). The metals investigated were Bi, Cr, Cu, Fe, Mg, Mn, Mo, Ni, Pb, V, and U. The operational conditions followed the 1994 United States Environmental Protection Agency (EPA) method entitled Laboratory Methods for ICP-MS Analysis of Trace Metals in Precipitation. This method covers all the necessary validation parameters for ICP-MS analysis, such as the operating conditions, instrument multi-element calibration standards, sensitivity checks, internal standard checks, and reagent blank.

The lower limits of detection were calculated as three times the standard deviation of the blank and were found to be 0.1, 225, 13, 436, 620, 17, 6, 43, 12, 16, and 2 ppb of Bi, Cr, Cu, Fe, Mg, Mn, Mo, Ni, Pb, V, and U, respectively. An independent quality control sample was used to certify all measurements in addition to the aforementioned standard reference material, the multi-element solution used originated from CertiPUR Merck No. OC528489. The percentage relative standard error for all measured metal concentrations was found to be 5.25%; this value represents the error on all metal concentrations reported unless otherwise specified.

### List of reagents

The reagents used to carry perform the analyses include; ultra-pure grade Nitric Acid (Scharlau Chemie S.A, Spain), pure grade Hydrogen Peroxide (Scharlau Chemie S.A, Spain), multi-element solution (CertiPUR Merck No. OC528489), Tealeaves (Certified reference no. 23, National Institute for Environmental Studies (NIES)), and Wheat flour (Certified reference no. 1567a, National Bureau of Standards (NIST)).

## Results and discussion

The distribution of the metal fractions in the separate compartments was similar in three of the four tobacco samples, with the exception of 06GRPBR, which had the lowest fraction of metal in the smoke. Concentration of all metals in the water vessel was low among all tobacco samples in comparison to the amount in the original tobacco (Table 3). Table 4 shows the concentrations of Bi, Cr, Cu, Fe, Mg, Mn, Mo, Ni, Pb, V, and U as an average of all the tobacco samples. Of all the metals, U appeared to have the largest fraction in the smoke compartment, while Mn was least available in the smoke. The effect of the metals on the users will depend not only on its availability in the smoke, but also on its toxicity and how readily it is absorbed into the body.

**Table 3:** **Total metal concentration (ppm) in four distinct sections of the waterpipe smoking process for each of the four waterpipe tobacco samples**

	**12LWMBR**	**13LWMBR**	**O6GRPBR**	**01APPRD**
Metal concentration in ppm ± standard deviation (faction of total in sample)				
Original sample	1653.49 ± 439.63	1434.51 ± 365.62	572.22 ± 117.71	2123.98 ± 482.14
	(1.0)	(1.0)	(1.0)	(1.0)
Ash residue	609.47 ± 144.25	473.45 ± 121.86	374.44 ± 97.32	492.72 ± 119.98
	(0.37)	(0.33)	(0.65)	(0.23)
Vater vessel	38.54 ± 11.19	52.98 ± 15.01	16.30 ± 3.96	54.65 **±** 15.38
	(0.02)	(0.04)	(0.03)	(0.03)
Smoke	1005.45 ± 286.33	888.86 ± 228.82	181.43 ± 33.37	1576.59 ± 348.39
	(0.61)	(0.62)	(0.32)	(0.74)
Of which >0.045 μm	87.95 ± 21.35	40.33 ± 11.56	55.76 *±* 9.64	45*.*25 *±* 8.72

The values represent the sum of all metals present (Bi, Cr, Cu, Fe, Mg, Mn, Mo, Ni, Pb, V, and U) with the standard deviation and the fraction of the total metal the value of each compartment represents.

**Table 4:** **Average metal concentrations (ppb) of four tobacco samples for each metal in four different compartments of the waterpipe setup**

**Metal concentration in ppb (fraction of total in sample)**					
**Metal**	**Original sample**	**Ash residue**	**Water vessel**	**Smoke**	**Of which >0.045 μm**
U	845.58 (1.00)	42.37 (0.05)	3.27 (0.004)	799.946 (0.95)	71.25
Cr	22656.66 (1.00)	4677.02 (0.21)	262.99 (0.012)	17716.645 (0.78)	781.51
Fe	212694.44 (1.00)	62577.98 (0.29)	2187.05 (0.010)	147929.416 (0.70)	1261517
Mg	1169370.72 (1.00)	404081.91 (0.35)	37931.91 (0.032)	727356.904 (0.62)	40906.88
Mn	21170.84 (1.00)	10903.92 (0.52)	35.48 (0.002)	10231.429 (0.48)	701.89
Mo	532.72 (1.00)	214.10 (0.40)	8.60 (0.016)	310.026 (0.58)	22.98
Pb	1329.66 (1.00)	449.00 (0.34)	37.47 (0.028)	843.188 (0.63)	294.56
Ni	10154.03 (1.00)	2216.33 (0.22)	78.52 (0.008)	7859.176 (0.77)	1159.21
V	578.94 (1.00)	170.58 (0.29)	25.23 (0.044)	383.127 (0.66)	29.50
Cu	6694.65 (1.00)	2179.41 (0.33)	46.84 (0.007)	4468.404 (0.67)	735.82
Bi	21.17 (1.00)	5.71 (0.27)	0.42 (0.020)	15.043 (0.71)	4.96

The fraction of metal in each compartment is calculated by dividing the average value of each metal from all samples in that compartment by the average value of that metal in the original sample. The readings are subject to an RSD of 5.25%.

For the purposes of analysing the total amount of metal waterpipe users are exposed to, the sum of the metal concentration in smoke and the amount accumulated on the filter were used. Exposure to the user may occur through smoke inhalation or mouth-level exposure. The typical waterpipe set-up does not include a filter. As a further analysis point, the amount on the filter is used to represent particle size of >0.45 μm (Figure 1). This pore size was selected according to the draft of the ISO/TC 126/SC standard, in which the particles have a diameter equal to or greater than 0.3 μm. The concentration of metal in the smoke and filter (total metal users are exposed to) was significantly larger than the concentration of metal remaining in the water vessel (Student’s t-test, *p-value* < 0.01), similarly the concentration of metal measured in the ash residue was also significantly larger than that in the water (Student’s t-test, *p-value* = 0.01) (Table 3). This demonstrates that the concentration of metal trapped in the water is significantly smaller in comparison to the total amount of metal present. This is further evidence in support of disproving the belief that the use of a waterpipe would protect from the harmful effects of tobacco smoking.

Of the total metal content in the four waterpipe tobacco samples tested, on average 40% ± 18% remained at the end of the smoking session as unavailable metal in the tobacco residue. A further 3% ± 1% remained as dissolved/suspended metal in the water. The remaining 57% ± 18% was the total exposure concentration, of which 5% ± 3% was of particle size 0.45 μm and above as this fraction was measured through the digestion of the filters (Figure 1).

This is in support of some trends reported in the literature, which state that the concentrations of toxicants (e.g. carbon monoxide or heavy metals) remain high even after bubbling through water [19]. As reported in a study by Shihadeh [20], there was no visible side-stream smoke during analysis however toxicants from the fuel may still be significant as second-hand smoke from waterpipes is a mixture of tobacco smoke and smoke from the fuel [1].

The actual amount of metal that is available for exposure through waterpipe use, as a function of the total metal that is contained in the original waterpipe tobacco sample, is dependent of the identity of the metal. However in all cases for Bi, Cr, Cu, Fe, Mg, Mn, Mo, Ni, Pb, V, and U the concentration of metal ‘filtered’ out during the water bubbling stage was around 3% (±1%) of the total metal originally contained. Therefore, it can be concluded that the small size of this fraction would not provide protection from potentially toxic metals. This finding is also true when comparing a number of different commercially available waterpipe tobaccos where origin, flavour, and colour do not alter the insignificance of the concentration of total metal retained in the water. For future analysis of heavy and essential metals in waterpipe tobacco, the need for including a number of compartments may not be required. Instead, it can be deduced that simply calculating the difference between the amount of total metal present in the tobacco sample and the amount remaining in the ash residue, possibly with the inclusion of a 3% (±1%) correction, will give a reasonable representation of the metal exposure.

A previous study has shown that waterpipe tobacco use exposes users to carcinogenic elements, such as U [10,15]. In this study, U was also found to be the most available metal in the smoke. Of the remaining metals that were readily available in the smoke, Ni is also known as a carcinogen, immunotoxic, and neurotoxic [21]. Chronic, long-term exposure to heavy metals has been known to increase the incidence of head and neck cancer in humans, with higher concentrations of Cr and Ni found in tumour tissues than in healthy tissue [22].

As previously mentioned, the results of the different tobacco samples used were averaged in order to give an overall idea of the amount of total metal available to users. However, given the wide variety of waterpipe tobacco available and the different methods of use, for example the mechanism set-up or the use of liquids other than water, it may be possible for the degree of exposure to differ. This could potentially be a limitation of the findings in this study. In addition, given that metal accumulation in tobacco leaves is a function of the geographic origin, generalisations with globally available waterpipe tobacco products must be considered with care. Indeed, a study by Saadawi et al. [23] found clear distinctions in the metal concentrations between waterpipe tobacco samples from the United States and those from the Middle East.

Currently, there are no specific health warning labels for waterpipe tobacco products that are approved by the WHO. A study based in Lebanon found that on the majority of waterpipe tobacco products, the health warning labels on average only covered 3.5% of the total surface area of the package [24]. Given the risks associated with human exposure to heavy metals and the high percentage of the total metal available to be transferred to the user, the use of adequate health warning labels is vital on all waterpipe tobacco products. At present, the waterpipe tobacco industry operates without regulation [25] and the impact of health warning labels on waterpipe use has not been extensively investigated. A recent preliminary study on regular waterpipe smokers in London, aimed to gauge the effectiveness of warning labels. Their initial findings suggested that waterpipe tobacco health warnings may be effective in changing smoking behaviour [26]. Overall, a larger commitment to stop waterpipe smoking is observed in individuals who are aware of the potential health concerns than in those who are not [25]. It is therefore essential that regulators and policymakers prioritise the correct labelling of waterpipe tobacco products in order to ensure users are informed of the dangers.

## Conclusion

The aim of this study was to quantitatively determine if the water filtration stage of the waterpipe smoking process successfully decreases exposure to heavy metals. For Bi, Cr, Cu, Fe, Mg, Mn, Mo, Ni, Pb, V, and U the percentage of metals removed during the water bubbling stage was around 3% (±1%) of the total metal. It was concluded that this small fraction would not protect the user against exposure to the majority of potentially toxic metals.

## References

[1] World Health Organization (Tobacco Free Initiative). TobReg Advisory Note: Waterpipe Tobacco Smoking: Health Effects. Research Needs and Recommended Actions by Regulators. WHO: Geneva, Switzerland; ISBN: 9241593857; 2005.

[2] Chaouachi K (2009). Hookah (Shisha, Narghile) Smoking and Environmental Tobacco Smoke (ETS).

[3] Maziak W, Eissenberg T, Ward K.D. Patterns of waterpipe use and dependence: implications for intervention development. Pharmacol Biochem Behav. 80:173–179. doi:10.1016/j.pbb.2004.10.026. 10.1016/j.pbb.2004.10.026 15652393

[4] Koul PA, Hajni MR, Sheikh MA, Khan UH, Shah A, Khan Y (2011). Hookah smoking and lung cancer in the Kashmir valley of the Indian subcontinent. Asian Pac J Cancer Prev.

[5] Alloway BJ. Heavy Metals in Soils. Blackie Academic and Professional: London, UK; 2nd edition, 1995.

[6] Golia EE, Dimirkou A, Mitsios IK (2009). Heavy‐Metal Concentration in Tobacco Leaves in Relation to Their Available Soil Fractions. Commun Soil Sci Plant Anal.

[7] Clemens S (2006). Toxic metal accumulation, responses to exposure and mechanisms of tolerance in plants. Biochimie.

[8] Shihadeh A, Saleh R (2005). Polycyclic aromatic hydrocarbons, carbon monoxide, “tar”, and nicotine in the mainstream smoke aerosol of the narghile water pipe. Food Chem Toxicol.

[9] Eissenberg T, Shihadeh A (2009). Waterpipe Tobacco and Cigarette Smoking: Direct Comparison of Toxicant Exposure. Am J Prev Med.

[10] Khater AEM, Abd El-Aziz NS, Al-Sewaidan HA, Chaouachi K (2008). Radiological hazards of Narghile (hookah, shisha, goza) smoking: activity concentrations and dose assessment. J Environ Radioact.

[11] Daher N, Saleh R, Jaroudi E, Sheheitli H, Badr T, Sepetdjian E (2010). Comparison of carcinogen, carbon monoxide, and ultrafine particle emissions fromnarghile waterpipe and cigarette smoking: Sidestream smoke measurements and assessment of second-hand smoke emission factors. Atmos Environ.

[12] Sepetdjian E, Saliba N, Shihadeh A (2010). Carcinogenic PAH in waterpipe charcoal products. Food Chem Toxicol.

[13] Maziak W, Rastam S, Shihadeh AL, Bazzi A, Ibrahim I, Zaatari GS (2011). Nicotine exposure in daily waterpipe smokers and its relation to puff topography. Addict Behav.

[14] Schubert J, Hahn J, Dettbarn G, Seidel A, Luch A, Schulz TG (2011). Mainstream smoke of the waterpipe: Does this environmental matrix reveal as significant source of toxic compounds?. Toxicol Lett.

[15] Al-Kazwini A, Said AJ, Al-Kharouf S (2012). Determination of natural and artificial radionuclide materials in the Massel used in Hubbly-Bubbly (Shisha) in Jordan. Toxicol Environ Chem.

[16] Al-Kazwini A, Sdepanian S, Said AJ (2014). Determination 1 of Macro and Trace Elements in Moassl Used in Waterpipe in Jordan. Publ Health Res.

[17] Shihadeh A, Salman R, Jaroudi E, Saliba N, Sepetdjian E, Blank MD (2012). Does switching to a tobacco-free waterpipe product reduce toxicant intake? A crossover study comparing CO, NO, PAH, volatile aldehydes, “tar” and nicotine yields. Food Chem Toxicol.

[18] Gatrad R, Gatrad A, Sheikh A (2007). Hookah smoking. Br Med J.

[19] Sajid KM, Akhter M, Malik GQ (1993). Carbon monoxide fractions in cigarette and hookah (hubble bubble) smoke. J Pakistan Med Assoc.

[20] Shihadeh A (2003). Investigation of mainstream smoke aerosol of the argileh water pipe. Food Chem Toxicol.

[21] Das KK, Das SN, DhundasiIndian SA (2008). Nickel, its adverse health effects and oxidative stress. Indian J Med Res.

[22] Khlifia R, Olmedoc P, Gilc F, Hammamid B, Chakround A, Rebaib A (2013). Arsenic, cadmium, chromium and nickel in cancerous and healthy tissues from patients with head and neck cancer. Sci Total Environ.

[23] Saadawi R, Figueroa JAL, Hanleya T, Caruso J (2012). The hookah series part 1: total metal analysis in hookah tobacco (narghile, shisha) – an initial study. Anal Meth.

[24] Nakkash R, Khalil J (2010). Health warning labelling practices on narghile (shisha, hookah) waterpipe tobacco products and related accessories. Tob Control.

[25] Jawad M, McEwen A, McNeill A and Shahab L. Waterpipe tobacco smoking. In: Preston A, McIlvar M, McEwen A, editors. National Centre for Smoking Cessation and Training 2013. Available online: http://www.ncsct.co.uk/usr/pub/waterpipe_briefing.pdf (last retrieved 12^th^ November 2014)

[26] Ali M, Grant A, Bakir A, Jawad S and Jawad M. Impact of waterpipe tobacco pack health warnings on waterpipe smoking behaviour: protocol and preliminary findings. UK National Smoking Cessation Conference: London, UK; 2014.

